# Recto-Vesico-Vaginal Cloaca

**Published:** 1912-09

**Authors:** 


RECTO-VESICO-VAGINAL CLOACA. Morestin reports a case of Potel, *Le Progres Med.,* Feb. 24, 1912, due to necrosis and infection following protracted instrumental labor in a primipara. Pauchet reports a congenital case in a girl of 16, without anus or urethra but with a well developed vulvar sphincter (though not sufficient to provide perfect continence. This case was also peculiar in that the single vulvar orifice led to a double vagina into each of which a uterine neck projected. Operative results were good in both cases. In the latter, after freeing the rectum, the posterior third of the vulvar sphincter was utilized as a sphincter ani. At a second operation, a musculo-mucous bridge was partially separated from the region of the vestibule and, beneath this, the neck of the bladder was implanted. The fæcal continence was excellent urinary control good by day but with leakage at night.

